# Opinions, Attitudes, and Hesitancy Concern the Vaccination for COVID‐19 Among Students of ‘Alexander Xhuvani’ University, Elbasan

**DOI:** 10.1002/nop2.70686

**Published:** 2026-07-25

**Authors:** Erjona Abazaj, Gjergji Koja, Lila Shundi, Zahide Sulejmani, Ela Ali, Brunilda Hysaj, Ledina Nikolla, Shpetim Qyra

**Affiliations:** ^1^ University ‘Alexander Xhuvani’ Elbasan Albania; ^2^ Institute of Public Health Tirana Albania; ^3^ Faculty of Technical Medical Science University of Medicine Tirana Albania

## Abstract

**Aim:**

We investigated the opinions, attitudes and hesitancy concerning the vaccine against the COVID‐19 virus among students at the university.

**Design:**

A descriptive cross‐sectional study.

**Methods:**

This is a cross‐sectional and descriptive survey conducted at a university from October to November 2021. Being at least 18 years old until 28, regardless of gender, being a student in one of the six programs at FTMS, and agreeing to participate in this investigation and to respond to our questionnaire were the inclusion criteria for this study. The outcome measures were the percentage of people agreeing to be vaccinated, opinions, attitudes, and factors and reasons linked with vaccination reluctance toward COVID‐19. The SPSS software was used to examine the data. ANOVA, *t*‐test, and *χ* ^2^ were performed to evaluate descriptive statistics, while logistic regression was used to find acceptance‐influencing variables. Two‐tailed tests were run, and a *p*‐value of less than 0.05 was deemed statistically significant.

**Results:**

Overall, of 242 participants, 74% were female, with a significant difference from males with a *p* = 0.02. About 66.1% of students identified the benefits and efficacy of controlling the disease, while 33.9% were not up to date on their vaccinations and thought that vaccinations could cause problems in the future. Students who did not want to be vaccinated were 27.7%. The results of this detailed study show skepticism concerning the COVID‐19‐ vaccine among students of the university, even though the rate of vaccination resulted in 60.74%. All findings suggest that public education on the efficacy and safety of the COVID‐19 vaccine is important for the future widespread use of the vaccine.

**Patient or Public Contribution:**

In this study, we involved the public contribution to understand their opinions, attitudes, and hesitancy concerning the vaccination for COVID‐19.

## 1 Introduction

A virus called coronavirus disease 19 caused a pandemic at the beginning of the year 2020 with a high impact on public health (Bai et al. 2021). Due to the lack of knowledge about the virus, its transmission mode, the high number of hospitalizations and mortality, and the lack of protective measures, treatment medications (Koja and Abazaj 2023) or protective vaccines in its beginnings caused a lot of uncertainty at the global level. Nowadays, it has been over 2 years since the first reported cases of COVID‐19 disease were identified in Wuhan, Hubei, China, and everything has changed. It is worth emphasizing the measures taken in the prevention of the virus and its management.

Fortunately, the successful development of medicine provided a lot of help in the treatment of COVID‐19, but the most cost‐effective preventive measure, obviously, is the treatment directly through the vaccine (Carlucci et al. 2020; Lurie et al. 2020). Over decades, vaccines have played a significant role in preventing infectious disease epidemics, and they are considered irreplaceable, especially for humans (Harrison and Wu 2020). During the arduous year of the epidemic, vaccine production was viewed as a ray of hope for all systems, particularly the school system. The reason for this was that after a long period of transitioning to online learning, all educational personnel and students or students might return to the auditoriums after receiving the COVID‐19 immunization.

On August 23, 2021, the U.S. Food and Drug Administration approved the first COVID‐19 vaccine. This vaccine has been known as the Pfizer‐BioNTech COVID‐19 Vaccine, and it was used for the prevention of COVID‐19 disease in individuals 16 years of age and older (FDA 2021). Over time many other vaccines have been approved by the FDA for widespread use. The data from June 10, 2021, showed that from 287 candidate vaccines, approximately 180 vaccines were used in preclinical studies, and over 65 of them were involved in direct medical development (World Health Organization 2021b; Jiang et al. 2022; MCVT 2020; Siqueira et al. 2022). The invention of the vaccine started as a research campaign in many countries throughout 2021 (Fontanet and Cauchemez 2020; Ashby and Best 2021), and as a consequence, these vaccines are available and considered to be safe and effective. SARS‐CoV‐2 vaccines in front of new virus variants still exist (Our World in Data 2021; ECDC 2021; Hodgson et al. 2021). Despite the development of the successful SARS‐COV‐2 vaccine, vaccine hesitancy and skepticism are becoming a threat to public health (Lin et al. 2020; Cvjetković et al. 2022).

Based to World Health Organization (2021b) they are vaccinated worldwide with one dose, only 67.2% of the population, with total complete series of 63.6% of the population (World Health Organization 2021b). In the Albanian population, uptake with at least one dose was 46.8%, uptake with a complete vaccine was 44.1% and uptake with an additional dose was 11.9%. Kim et al. (2020) in their paper, have determined the significant factors of hesitation or negative attitude concerning vaccines, which have caused misinformation based on social media influenza (Kim et al. 2020).

According to the hesitation or skepticism concerning the vaccine, the Ministry of Health, together with the whole health system, has carried out awareness campaigns regarding vaccination and mass media. However, there is high COVID‐19 vaccination hesitancy among the population globally including students. For all reasons, we investigated the opinions, attitudes, and hesitancy concerning the vaccine against the COVID‐19 virus among students of the university.

## 2 Methods

### 2.1 Research Context

The primary approach of this study involves the utilization of a cross‐sectional design, which, by its very nature, imposes inherent constraints on the capacity to derive causal inferences concerning the relationships between variables. The design under consideration provides a concise representation of the data at a specific moment in time. While it facilitates the examination of associations, it does not encompass the dynamic processes that may emerge over extended periods.

This cross‐sectional and descriptive survey study was conducted on university students (242 in total) from October to November 2021. This university was chosen because of the importance of this institution in the country. This university is an institution of higher education in Albania, founded in 1971 under the name Higher Pedagogical Institute, while in 1991, in the framework of the law on university autonomy, it was officially known as a public university.

The university is located in the city of Elbasan, which lies in central Albania. The geographical position of this city is quite strategic as it serves as a connecting point between the northern and southern parts of the Albanian lands. In the country, there are 15 public universities and many private Albanian universities, most of which are located in the country's capital. This is one of 6 universities in the district.

In this university, there are five faculties with many study programs, which have contributed to a high number of students. In this university, there are five faculties, the most important of which is the Faculty of Technical Medical Sciences, with several study programs, where the Nursing program has the highest number of students. The students who study at this university come mainly from the city of Elbasan, as well as the districts close to it. Today, the University of Elbasan has about 3500 students in the full‐time system and 4500 students in the part‐time system. It ranks tenth among public and private universities in the country in terms of its reputation and high‐quality teaching. Academic standards and the quality of the students' learning experience have been judged to be high, and the university's entry grade point average is a minimum of 6.50.

### 2.2 Recruitment

Before the study's commencement, in a meeting with the dean of the Faculty of Technical Medical Science (FTMS) and the leaders of the University, we explained the study's concepts, goals, and purposes and obtained the study's consent. Ethical clearance was obtained from the rectorate of the University. Following the leaders' consent, we extracted all of the students' emails with the assistance of the secretariat personnel, and we got in touch with each of them. Need to mention that every student at the Faculty of Technical Medical Sciences, where this survey was conducted, has a personal email address with which they can access the faculty site. Each student separately received an invitation email to participate in this study, along with comprehensive study details. Students in the invitation letter were informed that participation would be entirely voluntary, and they would be free to leave the study at any moment.

All of the data they will submit in the study's questions would be anonymous, and the working group would only use the data for this particular study discreetly. No personal data was collected except for gender, faculty, residence, study program, and so forth. The students were also told that there would be no financial compensation for their involvement in the study and that no gift compensation was allowed. To each person who wished to participate in this study, an email was sent asking for their confirmation of participation at the end of the email. Informed consent was obtained from each of them.

The inclusion criteria in our study were: the students were at least 18 years old and up to 28 years old, regardless of gender, enrolled in one of the six programs at FTMS, and all students who accepted to participate in this investigation and responded to our questionnaire. The working group disseminated the questionnaire online upon acceptance of participation. Also, during this study, the fulfillment of the standards of the Declaration of Helsinki was ensured.

### 2.3 Sample and Study Design

**FIGURE 1:**
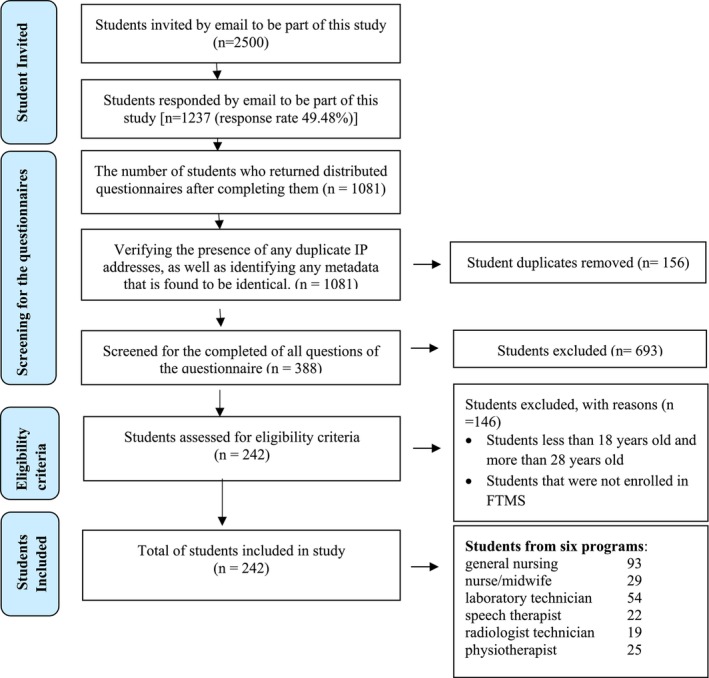
Flowchart for students' participation in the study.

The study population did not include all students of this University, but was limited to students from the Faculty of Technical Medical Sciences (FTMS). During the study period, 2500 students were enrolled in six academic programs on the faculty's premises (Figure 1). The single proportion calculation was used to determine the sample size (Degu 2005):

$$n=\frac{Z^{2}P\left(1-P\right)}{d^{2}}$$

where *n* = Sample size; *Z* = *Z* statistic for a level of confidence (1.96 for 95% confidence level); *P* = expected prevalence or proportion (20%); *d* = Precision (5%).

The invitation was sent to each FTMS student's email, but out of 2500 students, the participation rate resulted in approximately 50% (1237/2500) participating in this study. The questionnaire was distributed to various groups and programs. Initially, it was implemented for Bachelor's students, and subsequently, for Master's degree students, based on their course of study. Following its completion with the students in one program, it was carried over into the other programs. Owing to the brief duration of the study, only one reminder email was sent 5 days after the initial email was distributed.

The distribution of the 1237 questionnaires was conducted electronically via email, utilizing the acceptance rate of approximately 50% (1237/2500) of the students who had consented to participate in the study. The tools for completing the questionnaires were different when respondents were permitted to use a computer, tablet, mobile phone, or other communication tools. In the final analysis, 87.4% (1081/1237) of the distributed questionnaires were returned and subsequently evaluated by an individual specializing in computer science (IT specialist). This specialist assessed the IP addresses utilized or the use of identical metadata for each variable. Following a thorough examination of the IP and identical metadata, 14.4% (156/1081) of the questionnaires that had been completed on multiple occasions by the same student were excluded from the analysis.

Subsequently, the responsibility for the management of the downloaded questionnaires was given to two specialists (a medical staff member and a vaccinator). The aforementioned individuals were also tasked with the evaluation of each questionnaire with a view to ascertaining the manner in which each of the questionnaire items had been answered. Following a rigorous examination, it was determined that for each component, no more than two items that remained unanswered should be considered inconsistent. Following a detailed analysis of each of the questionnaires, it was decided that approximately 35.9% (693/1081) of them were to be excluded from the final statistical analysis due to incorrect completion or lack of completion.

Following this, a second group was assigned to evaluate the questionnaires regarding the eligibility criteria for each of the participating students. Subsequent to the implementation of the control, it was ascertained that 62.4% (242/388) of the students, or approximately 22.4% of the total number of FTMS students who fulfilled and returned the questionnaire, were to be taken into consideration for statistical evaluation regarding the purpose and objectives of this study.

The subsequent flowchart illustrates the method by which data were collected from participants in the present study (Figure 1). Because sufficiently detailed auxiliary information was not available for all invited students, a weighting‐based correction for non‐response could not be performed. The final sample was therefore described in detail, and the findings were interpreted with caution in view of the potential for selection bias related to differential participation.

### 2.4 Data Collection Procedure

The initial focus of the working group following the study's conceptualization was on data collection. Prior studies focused on ascertaining the opinions, attitudes and hesitations of various studies toward vaccines, including those against the novel strain of SARS‐CoV‐2.

Following the collation of a range of studies, the working group designed a questionnaire based on the findings of earlier research. Following an extensive analysis of all available findings, it was initially concluded that questions would be extracted from studies demonstrating similar methodologies (Serbezova et al. 2021; Usman et al. 2021; Bălan et al. 2021; Sadaqat et al. 2021; Barello et al. 2020; Kaya et al. 2021). In the works of Serbezova et al. (2021), Usman et al. (2021), and Bălan et al. (2021), questions pertaining to knowledge, acceptability, attitude, and concern regarding the vaccination against the virus were extracted. In contrast, the works of Barello et al. (2020) and Sadaqat et al. (2021) focused on the extraction of questions related to hesitancy toward the aforementioned vaccination. Following the extraction of the questions, the working group selected the questions based on the purpose and objectives that were set for this study on the receipt of the vaccine for the prevention of the novel strain of coronaviruses among Albanian students. It was also imperative to ensure that the questions were closely aligned with the cognitive processes and conceptual frameworks of our students.

The initial questionnaire was drafted in English. Subsequently, a translation into Albanian was conducted by the working group. Subsequently, the translation was subjected to a rigorous review process, during which it was revised by a licensed translator. Following standardization and adaptation of the questionnaire, the standardization of the questionnaire was facilitated. The standardized questionnaire employed in this study was characterized by elevated readability and comprehensibility, which was predicated on the levels of the study participants. The self‐administered questionnaire, which comprised closed questions, was designed to be completed within a maximum timeframe of 30 min, with the result that Cronbach's alpha for the questionnaire was 0.835.

### 2.5 Study Instrument

The dependent variable was students' attitudes toward the administration of a vaccine for the novel pathogen SARS‐CoV‐2, while as the independent variables, the following elements were considered: (1) The demographic variables (considered included: age, gender, place of residence and degree); (2) The vaccination status of students, along with their preferences regarding vaccine types, was also examined; (3) In addition, the knowledge, beliefs, and risk perceptions concerning the pandemic and vaccines were analysed; (4) Finally, the hesitation and concerns of students concerning the vaccination were investigated. The questionnaire consisted of 44 questions, organized into five components, each comprising numerous items.

### 2.6 Measurement of Variables

#### 2.6.1 Demographic Variables (Independent Variables)

The demographic characteristics component was used to collect individual student data. This component was conceptualized into seven distinct items, encompassing inquiries pertaining to the age (years) (18–21 = 1; > 21–25 = 2); gender (male = 0; female = 1); geographical location city (city of Elbasan = 0; others = 1); residence area (urban = 0; rural = 1); place of residence (dormitory = 0; life with family = 1; life with friends = 2); academic programmes degree (Bachelor's = 0; Master's = 1); study programmes (general nursing = 0; nurse/midwife = 1; laboratory technician = 2; speech therapist = 3; radiologic technician = 4; physiotherapist = 5). The inclusion of these variables was informed by preliminary evidence that suggested a potential influence on attitudes toward vaccination. City of origin was retained in the multivariable analysis because students came both from Elbasan and from other Albanian cities, and this variable was considered a potentially relevant contextual factor related to vaccine attitudes. A range of other independent variables has been identified as being associated with levels of acceptance of the vaccine, levels of knowledge, degrees of hesitation, and perceptions (Table 1).

**TABLE 1:** Internal consistency of the scale in the present study (*N* = 242).

Components	Variables		Number of items	Cronbach's alpha
Component 1	Independent variables	Demographic variables	7	0.770
Component 2		Vaccination and vaccine preference types	6	0.810
Component 3		Knowledge, beliefs, and risk perception concerning the COVID‐19 pandemic and vaccines	13	0.812
Component 4		Hesitation and concerns of students about the COVID‐19 vaccine	8	0.894
Component 5	Dependent variable	Attitude and concern of students with the COVID‐19 vaccine	10	0.90

#### 2.6.2 Vaccination and Vaccine Preference Types Variables (Independent Variables)

In order to ascertain the vaccination rate of students at the time of the study, the students' vaccination status was analysed, and the types of vaccine for which there was a preference were recorded. These questions are of a closed nature, with each student required to select one of the provided alternatives. The questionnaire comprised dichotomous questions (answered ‘no’ or ‘yes’), and multiple‐choice questions with more than two possible answers. The present component was designed based on six items, and following statistical analysis, the instrument demonstrated acceptable internal consistency in the current study (overall Cronbach's *α* = 0.770) (Table 1).

#### 2.6.3 Variables for Knowledge, Beliefs, and Risk Perception Concerning the COVID‐19 Pandemic and Vaccines (Independent Variables)

The study utilized an empirical approach to assess students' comprehension of the vaccine, their perceived credibility, and the risks or concerns associated with it. This assessment was informed by their knowledge, beliefs, and risk perceptions concerning the pandemic and vaccines. The component under consideration consists of 13 items, which are dichotomous in nature (no = 0 and yes = 1). In the current sample, the Cronbach's alpha scale demonstrated adequate reliability (*α* = 0.812) (Table 1).

#### 2.6.4 Variables for Hesitation and Concerns of Students About the COVID‐19 Vaccine (Independent Variables)

In the fourth domain, we measure the degree to which students were hesitant to get vaccinated. The component under consideration consists of 8 items, which are dichotomous in nature (no = 0 and yes = 1). In this instance, the Cronbach's alpha scale demonstrated excellent reliability, with a value of *α* = 0.894 (Table 1).

#### 2.6.5 Variables for Attitude and Concern of Students With the COVID‐19 Vaccine (Dependent Variable)

The perception of risk and beliefs or attitudes regarding COVID‐19 vaccines against the pandemic constituted the dependent variable in this study. This component is designed in 10 items, and the assessment was made based on Likert's scale assessments, ranging from 1 = Strongly Disagree, 2 = Disagree, 3 = Neutral, 4 = Agree, and 5 = Strongly Agree. The sub‐scales can be used individually and were calculated by averaging the item responses, with higher scores indicating a stronger presence of attitude and concern. A higher score indicated a stronger attitude and concern toward the COVID‐19 vaccine during the pandemic. There is no cut‐off point on the scale. The findings of the scale analysis on the attitude and concern with COVID‐19 vaccine variables were excellent (overall Cronbach's *α* = 0.90) (Table 1). For the primary regression analysis, the ordered nature of the attitude scale was retained. For secondary analyses, a dichotomised attitude outcome was created by classifying students with a mean attitude score of < 3.0 as having a negative attitude and those with a mean score of ≥ 3.0 as having a positive/neutral attitude; the reference category was defined consistently in all regression tables.

### 2.7 Questionnaire Piloting

Prior to the official distribution of the aforementioned questionnaire, an assessment of its feasibility, acceptability and understandability was conducted with 45 students who met the inclusion criteria for the study. This phase was utilized for the purpose of evaluating the technical functionality of the survey instrument within the context of the study. It also encompassed the assessment of the time required to complete each question and the suitability of the question wording in the cultural and educational context of the participants for each of the questionnaire domains. The data collected during this piloting phase were excluded from the final sample analysis. Following a thorough evaluation of the responses collected during the initial piloting phase, minor adjustments were made to specific questions. It is important to note that no substantial changes were made to the content, structure, or scoring of the standardized instruments. This is because they demonstrated strong conceptual clarity and psychometric validity. Following the implementation of these adjustments, a second pilot phase was conducted, involving 30 students, whose responses were included in the final report. In this phase, positive feedback was received from all participants in the second pilot programme, who indicated that each question was well‐written and easily comprehensible. In conclusion, it was decided that the questionnaire would be completed by each of the students invited to participate in this study, so the email addresses of the users were not limited.

### 2.8 Ethical Considerations

This study was approved by the Ethics Committee, University of Medicine, Tirana, approval number 523/2 dated 15.09.2021. All procedures were conducted in accordance with the Declaration of Helsinki. Written informed consent was obtained from all participants before completion of the questionnaire. Students were informed that participation was voluntary, anonymous, and that they could withdraw at any time without any academic or personal consequences. No financial or material incentives were provided, and no directly identifying personal information was collected.

### 2.9 Statistical Analyses

Data were analysed using IBM SPSS Statistics version 30.0 (IBM Corp., Armonk, NY, USA). Descriptive statistics are presented as frequencies and percentages for categorical variables and as mean ± standard deviation or median (interquartile range), as appropriate, for continuous variables. The primary attitudinal outcome was derived from the 10‐item attitude scale. Because this outcome originated from Likert‐scale items, the main multivariable analysis used an ordinal logistic regression model in order to preserve the ordinal structure of the response. In a secondary analysis, the attitude score was dichotomized using a prespecified threshold, whereby a mean attitude score of < 3.0 defined a negative attitude and a mean score of ≥ 3.0 defined a positive/neutral attitude and was analysed using binary logistic regression for comparability with the originally reported findings.

The multivariable model was specified a priori on theoretical and contextual grounds rather than being selected solely according to univariable *p*‐values. Variables considered for inclusion were sex, age group, city of origin, residence area, living arrangement, degree level and study programme, as these were considered conceptually relevant to attitudes toward COVID‐19 vaccination. Multicollinearity was assessed using variance inflation factors (VIFs). For the binary logistic model, calibration was evaluated using the Hosmer–Lemeshow goodness‐of‐fit test, explanatory performance was summarized using pseudo‐*R* ^2^, and discrimination was assessed using the area under the receiver operating characteristic curve (AUC). Adjusted odds ratios (aORs) with 95% confidence intervals (95% CIs) are reported. All tests were two‐sided, and *p* < 0.05 was considered statistically significant. As a sensitivity analysis, we repeated the binary logistic regression using alternative thresholds for the dichotomized attitude score. In the main analysis, a mean attitude score of < 3.0 defined negative attitude. Sensitivity analyses additionally used a stricter threshold of < 2.5 and a more inclusive threshold of < 3.5. The same covariates were retained in all models to allow direct comparison across specifications. As an additional sensitivity analysis, we repeated the main regression model after excluding students who reported that their vaccination was primarily motivated by mandatory requirements from the government or faculty. This analysis was undertaken to evaluate whether the observed associations were robust after removing participants whose vaccination behaviour may have been influenced predominantly by external mandates rather than personal attitudes.

To assess potential response or selection bias, we compared students included in the final analytical sample with students who were invited or initially agreed to participate but were not included in the final analysis. The comparison was performed using the available characteristics from the study records, including sex, age group, degree level, study programme and residence area. Categorical variables were compared using the chi‐squared test or Fisher's exact test, as appropriate. Because the questionnaire was anonymous and complete individual‐level information was not available for all invited non‐responders, the comparison was limited to variables available in the recruitment and questionnaire records.

Missing data were assessed before statistical analysis. For each study variable, the number and percentage of missing values were calculated and summarized. Questionnaires with duplicate metadata, extensive incompleteness, or failure to meet the eligibility criteria were excluded before construction of the final analytical dataset. Within the final analytical sample, missingness was evaluated separately for demographic variables, vaccination‐related variables, knowledge and risk‐perception items, hesitancy items, attitude items, and the main attitude outcome. Complete‐case analysis was used for the primary regression models. Scale scores were calculated only when the predefined valid‐response rule for the respective domain was met.

Multiple imputation was considered as a sensitivity approach for missing data. However, it was not applied to questionnaires excluded before construction of the final analytical dataset because these questionnaires had duplicate metadata, extensive incompleteness, or did not meet the eligibility criteria. In these cases, the main composite measures and the primary attitude outcome could not be validly constructed. In addition, because the survey was anonymous, sufficiently detailed auxiliary information was not available for all non‐responders or non‐included students to support reliable imputation. Therefore, the primary analysis was conducted using complete cases within the final analytical sample.

Exploratory factor analysis was conducted to assess the construct validity of the composite questionnaire domains. Principal axis factoring was used because the items were designed to reflect underlying latent constructs. Oblique rotation was applied because the domains of knowledge, beliefs, hesitancy, and attitudes were expected to be correlated. Sampling adequacy was assessed using the Kaiser–Meyer–Olkin statistic, and factorability was assessed using Bartlett's test of sphericity. Factors were retained based on eigenvalues greater than 1, scree plot inspection, and conceptual interpretability. Factor loadings ≥ 0.40 were considered meaningful. Internal consistency of the retained domains was assessed using Cronbach's alpha.

## 3 Results

Initially, a total of 2500 FTMS students were invited to participate in this study. Of those, a distribution of questionnaires was executed electronically via email only to 1237 students who had responded affirmatively to the invitation to participate, resulting in an approximate acceptance rate of 50%. The final return rate for the distributed questionnaires was 87.4%, with 1081 out of 1237 questionnaires returned. Following a thorough evaluation by Information Technology specialists to identify instances of duplication, and by field specialists to ascertain the completion or non‐completion of each question and the fulfillment of the inclusion criteria, it was determined that approximately 22.4% of the total number of FTMS students who fulfilled and returned the questionnaire were to be considered for statistical evaluation regarding the purpose and objectives of this study.

To assess the possibility of response or selection bias, we compared students included in the final analytical sample with students who were not included in the final analysis. The non‐included group comprised students who were invited or initially agreed to participate but were subsequently excluded because of non‐returned questionnaires, duplicate responses, extensive incompleteness, or failure to meet the eligibility criteria. The comparison was performed using the available demographic and academic characteristics recorded during recruitment and questionnaire screening. Because the survey was anonymous, a complete individual‐level comparison with all initially invited non‐responders was not possible; therefore, the analysis was limited to variables available in the study records (Table 2).

**TABLE 2:** Comparison between included responders and non‐included students using available characteristics.

Characteristics	Included responders *n* = 242	Non‐included students *n* = 995	*p*
Sex			
Male	63 (26.0)	342 (34.4)	0.012
Female	179 (74.0)	653 (65.6)	
Age group, years			
18–21	146 (60.3)	574 (57.7)	0.456
> 21–25	96 (39.7)	421 (42.3)	
Degree level			
Bachelor's	154 (63.6)	671 (67.4)	0.258
Master's	88 (36.4)	324 (32.6)	
Study programme			
General nursing	93 (38.4)	361 (36.3)	0.681
Nurse/midwife	29 (12.0)	134 (13.5)	
Laboratory technician	54 (22.3)	206 (20.7)	
Speech therapist	22 (9.1)	104 (10.5)	
Radiology technician	19 (7.9)	91 (9.1)	
Physiotherapist	25 (10.3)	99 (9.9)	
Residence area			
Urban	167 (69.0)	617 (62.0)	0.043
Rural	75 (31.0)	378 (38.0)	

*Note:* Values are presented as *n* (%). The non‐included group includes students who accepted participation but were not included in the final analytical sample because of non‐returned questionnaires, duplicate responses, extensive incompleteness, or failure to meet eligibility criteria. Comparisons were performed using the chi‐squared test.

A comparison between included responders and non‐included students was conducted using the available characteristics. Included responders were more frequently female and more often from urban areas compared with non‐included students. No statistically significant differences were observed by age group, degree level, or study programme.

Missing data were summarized for all variables included in the descriptive and analytical components of the study. Because questionnaires with extensive incompleteness were excluded before construction of the final analytical sample, the level of missingness within the final dataset was low. The missing‐data summary and the corresponding handling strategy are presented in Table 3.

**TABLE 3:** Missing data by study variable and handling strategy in the final analytical sample.

Variables	Eligible *N*	Missing *n*	Missing %	Handling strategy
Sex	242	0	0.0	Complete‐case analysis
Age group	242	0	0.0	Complete‐case analysis
City of origin	242	0	0.0	Complete‐case analysis
Residence area	242	0	0.0	Complete‐case analysis
Living arrangement	242	0	0.0	Complete‐case analysis
Degree level	242	0	0.0	Complete‐case analysis
Study programme	242	0	0.0	Complete‐case analysis
Vaccination status	242	1	0.4	Complete‐case analysis
Number of vaccine doses	242	2	0.8	Complete‐case analysis
Reason for vaccination	147	3	2.0	Analysed among vaccinated students only
Vaccine type received	147	2	1.4	Analysed among vaccinated students only
Vaccine preference among unvaccinated students planning vaccination	28	1	3.6	Analysed among eligible subgroup only
Previous COVID‐19 infection	242	1	0.4	Complete‐case analysis
Knowledge, beliefs and risk perception items	242	3	1.2	Scale score calculated when valid‐response rule was met
Hesitancy and concern items	242	4	1.7	Scale score calculated when valid‐response rule was met
Attitude and concern Likert‐scale items	242	5	2.1	Scale score calculated when valid‐response rule was met
Main attitude outcome	242	5	2.1	Complete‐case analysis in regression models
Sources of information	242	2	0.8	Multiple‐response descriptive analysis

*Note:* Values are presented for the final analytical sample. Questionnaires with duplicate metadata, extensive incompleteness, or failure to meet the eligibility criteria were excluded before construction of the final analytical dataset.

In the final analytical sample, demographic variables had no missing values, whereas vaccination‐related and questionnaire‐scale variables had low levels of missingness. Missingness for the main attitude outcome was limited and was handled using complete‐case analysis in the regression models. Variables applicable only to specific subgroups, such as reason for vaccination or vaccine type received, were analysed only among eligible participants. No variable had a level of missingness within the final analytical sample that suggested substantial loss of information after exclusion of extensively incomplete questionnaires.

Multiple imputation was evaluated but was not performed because the excluded questionnaires lacked sufficient valid item‐level information to construct the main composite domains and the primary attitude outcome. Within the final analytical sample, missingness was low and did not materially affect the number of observations available for the primary regression model. Therefore, the main analysis was conducted using complete cases. Because multiple imputation could not be validly implemented for the excluded questionnaires, no imputed regression estimates were generated for comparison with the complete‐case model.

Exploratory factor analysis was performed to evaluate the construct validity of the composite questionnaire domains used in the study. The analysis was applied to the domains containing multiple questionnaire items, including vaccination and vaccine preference, knowledge/beliefs/risk perception, hesitancy and concern, and attitude and concern toward the COVID‐19 vaccine. The results are summarized in Table 4.

**TABLE 4:** Exploratory factor analysis of the composite questionnaire domains.

Composite domain	Number of items	KMO	Bartlett's test *p*‐value	Retained factors	Total variance explained (%)	Range of main factor loadings	Cronbach's alpha
Vaccination and vaccine preference types	6	0.74	< 0.001	1	52.6	0.48–0.79	0.810
Knowledge, beliefs, and risk perception concerning the COVID‐19 pandemic and vaccines	13	0.82	< 0.001	2	58.4	0.44–0.83	0.812
Hesitancy and concerns about the COVID‐19 vaccine	8	0.86	< 0.001	1	61.7	0.57–0.88	0.894
Attitude and concern toward the COVID‐19 vaccine	10	0.88	< 0.001	2	64.2	0.51–0.86	0.900

*Note:* Exploratory factor analysis was conducted using principal axis factoring with oblique rotation. Factor retention was based on eigenvalues greater than 1, scree plot inspection, and conceptual interpretability. Factor loadings ≥ 0.40 were considered meaningful.

Abbreviation: KMO, Kaiser–Meyer–Olkin measure of sampling adequacy.

The exploratory factor analysis supported the empirical structure of the composite questionnaire domains. The Kaiser–Meyer–Olkin values ranged from 0.74 to 0.88, indicating acceptable to good sampling adequacy, and Bartlett's tests of sphericity were statistically significant for all domains. The vaccination preference and hesitancy domains showed a one‐factor structure, whereas the knowledge/risk perception and attitude/concern domains suggested two related factors. The retained factors explained between 52.6% and 64.2% of the total variance. Internal consistency was acceptable to excellent across domains, with Cronbach's alpha values ranging from 0.810 to 0.900.

Overall, 242 student participants in this study, most of them were females 74% (179/242), with a significant association with males. The average age resulted in 19.9 years ±1.8 SD with a minimum age of 18 years old and a max of 25 years old. The students in the age groups 18–21 years old show the highest number 60.3% (146/242) compared to the age groups > 21–25 years old 39.7% (96/242). A significant association was found between age groups. Additionally, 69% (167/242) lived in urban areas and 31% (75/242) in rural areas. Most of the students were from Elbasan city 65.29% (158/242) and other students 34.71% (84/242) are coming from different cities in Albania to be part of this university. Furthermore, one‐third of the students 24.4% (59/242) live in the dormitory during their studies, half of them 50.4% (122/242) live with their families, and the other 25.2% (61/242) share the house with friends or are displaced from their family environment. This university offers many study programs in different faculties, and the levels of programs are Bachelor's and Master's degrees. This study included only students from the Faculty of Technical Medical Sciences with studies programs like general nurses, nurses/Midwives, Laboratory and radiologist technicians, Speech therapists, and Physiotherapists in two degrees (Bachelor's and Master's levels).

Based on calculation data, 63.6% (154/242) of students were at the Bachelor's level and 36.4% were at the Master's level. According to the study programs, most of the participants, 38.5%, were general nurses (Table 5).

**TABLE 5:** Sociodemographic characteristics of students.

Variables		Frequency	Percentage	*p*
Gender	Male	63	26%	0.02
	Female	179	74%	
Age groups (years old)	18–21	146	60.3%	0.001
	> 21–25	96	39.7%	
City	Elbasan	158	65.29%	0.04
	Other	84	34.71%	
Residence area	Urban	167	69%	0.7
	Rural	75	31%	
Living place	Dormitory	59	24.4%	0.005
	Living with family	122	50.4%	
	Living with friends	61	25.2%	
Degree	Bachelor's	154	63.6%.	0.3
	Master's	88	36.4%	
Faculty of Technical Medical Science: study programs	General Nursing	93	38.40%	0.5
	Nurse/Midwives	29	12%	
	Laboratory technician	54	22.30%	
	Speech therapist	22	9.1%	
	Radiologist technician	19	7.90%	
	Physiotherapists	25	10.30%	

The status of vaccinated students is presented in Table 6. Students who had received almost one dose of the vaccine were 60.74% (147/242) of participants, and 39.26% (95/242) had applied the vaccine at the time we did this study. Approximately 27.7% (67/242) of students were categorically against taking the COVID‐19 vaccine, while other students who had not done it, 11.6% (28/242), have planned to do it as soon as possible in the following days. On the other hand, 40.9% (99/242) had received only one dose, and 19.8% (48/242) had two doses.

**TABLE 6:** Vaccination students, preference of the vaccine types.

Vaccination		Frequency	Percentage
Have you been vaccinated?	Yes	147	60.74%
	No	95	39.26%
How many doses have you done?	One dose	99	40.9%
	Two doses	48	19.8%
	Not yet vaccinated, but they plan to do	28	11.6%
	I do not want to be vaccinated	67	27.7%
The reasons that you have vaccinated yourself	Mandatory	104	70.7%
	To protect me and others	38	25.9%
	I have afraid of the COVID‐19 virus	5	3.4%
Does the type of vaccine influence your decision?	Yes	29	19.7%
	No	118	80.3%
What type of vaccine have you been vaccinated with?	Traditional vaccine/vector vaccine	97	66%
	RNA vaccine	50	34%
If you are not vaccinated yet but would like to be vaccinated in the next few days, which type would you like to be vaccinated with?	Traditional vaccine/vector vaccine	16	57.14%
	RNA vaccine	12	42.86%

Regarding the reasons for vaccinations with one of the types of COVID‐19 vaccines among 147 vaccinated students, about 70.7% (104/147) of them said that vaccination was mandatory by the government and faculty. Nearly 25.9% (38/147) reported that they have been vaccinated because they want to protect themselves and others from infection by the virus, and only 3.4% (5/147) of students referred to were afraid of the COVID‐19 virus. All students who have been referred for the vaccination are asked if the type of vaccine matters in their decision.

Based on the calculation data, only 19.7% (29/147) said yes, and the others said no, 80.3% (118/147). Furthermore, 66% (97/147) have been vaccinated with traditional vaccine/vector vaccine and 34% (50/147) with RNA vaccine. Meantime, 11.6% of the students reported that they were not vaccinated yet but would like to be vaccinated in the next few days. Most of them, 57.14% (16/28), preferred to be vaccinated with a traditional vaccine/vector vaccine, and the other 42.86% (12/28) with an RNA vaccine (Table 6).

Knowledge, beliefs, and risk perceptions concerning the pandemic and COVID‐19 vaccine among students are presented in Table 7. The results showed that 45% (109/242) of students had previously tested positive for COVID‐19. More than half, 56.6% (137/242), think that COVID‐19 should be mandatory for the general population, and 23.1% (56/242) mention that COVID‐19 will be managed only by precautionary measures without vaccination. Additionally, 66.1% (160/242) of students know the benefits and efficacy of vaccines in controlling the disease; meanwhile, 43.4% (105/242) think that after vaccination, they do not need to wear a mask in public or take other precautions. Almost 40.1% (97/242) of the students thought that vaccines caused problems in the future; on the other hand, 29.7% (72/242) of them stated that vaccines are safe. Meantime, about 25.2% (61/242) of students have mentioned that it does not make sense to get a vaccine at the time that we have passed COVID‐19, and 55.4% (134/242) think that natural protection is better than vaccination protection. In addition, 49.6% (120/242) of students think that vaccines were produced very quickly, and the side effects they will produce in the future are unknown (Table 7).

**TABLE 7:** Descriptive statistics for items measuring the knowledge, beliefs, and risk perceptions concerning the COVID‐19 pandemic and vaccines.

Variables (only answer yes)	Frequency	Percentage
Have you ever been infected with COVID‐19?	109	45%
Do you think that the COVID‐19 vaccine should be mandatory for people (especially risk groups)?	137	56.6%
Do you think COVID‐19 will be controlled only by preventive measures without vaccination?	56	23.1%
Do you think that various discovered COVID‐19 vaccines can protect from this disease?	153	63.2%
Is vaccination a safe and effective way to deal with a global pandemic?	160	66.1%
Do you think that vaccines are safe?	72	29.7%
We are afraid to believe that vaccines can cause infertility	97	40.1%
If I have had COVID it is not necessary to be vaccinated	61	25.2%
I will not wear a mask and take other precautions if I am vaccinated	105	43.4%
Natural protection is better than vaccination protection	134	55.4%
Vaccines can cause some mental health diseases	38	15.7%
Vaccines were produced very quickly and the side effects they will produce are unknown	120	49.6%
Vaccines permanently alter our DNA	46	19%

Related to the hesitation concerning the COVID‐19 vaccine, about 24% (58/242) have seen side effects such as rash, allergy, and high temperature in their relatives. Students were generally against mandatory vaccination at a young age, mainly under 25 years of age; this was referred to in 45% (109/242) of them, and 33.9% (82/242) think that this vaccine can have later effects on age. Almost 38.8% (94/242) of students think that they are young and can easily cope with the disease if they get infected. Students have hesitation related to the vaccine because 27.7% (67/242) of them do not have access to choose the type of vaccine. Only 9.5% (23/242) have referred that they suffer from other diseases and cannot get vaccinated, 4.5% (11/242) do not like to be pierced, and 34.7% (84/242) do not trust vaccines (Table 8).

**TABLE 8:** Descriptive statistics for items measuring the hesitation of students' concerns about the COVID‐19 vaccine.

Variables	Frequency	Percentage
This vaccine has demonstrated side effects such as rash, allergy and high temperature in our relatives	58	24%
We do not like that it has become mandatory for our age	109	45%
We think that it can have later effects on our age	82	33.9%
I am at a young age and I can easily cope with the disease if I get infected	94	38.8%
We have no access to choose the type of vaccine	67	27.7%
I suffer from other diseases and cannot get the vaccine	23	9.5%
I do not like to be pierced	11	4.5%
I do not trust vaccines	84	34.7%

The students are asked about the sources of information related to the pandemic and COVID‐19 vaccines. We need to mention that the information was coming from different sources. Students chose one or more alternatives. Based on what students have referred to, almost 64% (155/242) of the sources of information used mass media, most of them 72.7% (176/242) used social media/internet, 12% (29/242) heard information from friends, 38% (92/242) from medical staff, 14.9% (36/242) from the faculty and lecturer, and 44% (106/242) from the promotion of government, especially the Ministry of Health, since the beginning of the pandemic and application for the first time of the vaccine in our country.

Table 9 shows the attitude of students concerning the COVID‐19 vaccine by using the Likert scale assessments. Even though most of the students have a positive attitude concerning the vaccine, they show dissatisfaction. Based on the Likert's scale assessments, all the answers in neutral, agree and strongly agree are assessed as positive attitudes, and the answers to strongly disagree and disagree are assessed as negative attitudes concerning the vaccines (Table 9).

**TABLE 9:** The attitude of students concerned with the COVID‐19 vaccine/Likert's scale assessments.

Questionnaires	Likert's scale	Value	
		*N*	%
The vaccines, currently given in Albania are the same that those vaccines innovative countries are taking	Strongly disagree	24	9.9
	Disagree	48	19.83
	Neutral	56	23.14
	Agree	78	32.23
	Strongly agree	36	14.9
The persons who take the COVID‐19 vaccination, have created immunity by helping others	Strongly disagree	51	21
	Disagree	82	34
	Neutral	65	26.85
	Agree	27	11.15
	Strongly agree	17	7
I was very optimistic about taking the vaccine	Strongly disagree	75	31
	Disagree	44	18.18
	Neutral	48	19.83
	Agree	41	16.94
	Strongly agree	34	14.05
I motivated others to take the vaccine	Strongly disagree	73	30.16
	Disagree	63	26.03
	Neutral	31	12.81
	Agree	58	24
	Strongly agree	17	7
COVID‐19 cannot be controlled without vaccination	Strongly disagree	56	23.14
	Disagree	44	18.18
	Neutral	100	41.32
	Agree	27	11.16
	Strongly agree	15	6.2
The COVID‐19 vaccine is fairly distributed to all	Strongly disagree	78	32.23
	Disagree	39	16.12
	Neutral	88	36.36
	Agree	27	11.16
	Strongly agree	10	4.13
Vaccination absolutely prevents the virus	Strongly disagree	22	9.09
	Disagree	31	12.81
	Neutral	92	38.02
	Agree	61	25.2
	Strongly agree	36	14.88
The surest way to fight the virus is through vaccination	Strongly disagree	58	24
	Disagree	90	37.19
	Neutral	41	16.94
	Agree	24	9.9
	Strongly agree	29	11.97
The COVID‐19 vaccine is not tested potentially for its effectiveness	Strongly disagree	90	37.19
	Disagree	87	35.95
	Neutral	36	14.88
	Agree	22	9.09
	Strongly agree	7	2.89
The non‐subjective attitude concerns the virus	Strongly disagree	22	9.09
	Disagree	58	24
	Neutral	78	32.23
	Agree	60	24.78
	Strongly agree	24	9.9

Table 10 presents the theory‐based multivariable logistic regression model for factors associated with negative attitudes toward COVID‐19 vaccination. In the adjusted model, residence area and living arrangement showed the most consistent associations with negative attitudes toward COVID‐19 vaccination. Students from rural areas had higher odds of a negative attitude compared with students from urban areas (aOR = 2.64, 95% CI 1.51–4.61, *p* = 0.001). Students living in dormitories also had higher odds of a negative attitude than those living with family (aOR = 1.82, 95% CI 1.03–3.24, *p* = 0.039), whereas the association for students living with friends was weaker and did not reach statistical significance (aOR = 1.41, 95% CI 0.76–2.63, *p* = 0.271). Female students had higher adjusted odds of a negative attitude compared with male students, although this association was imprecise (aOR = 1.67, 95% CI 0.96–2.91, *p* = 0.071). Similarly, age group, city of origin and degree level were not independently associated with the outcome after adjustment.

**TABLE 10:** Theory‐based multivariable logistic regression model for factors associated with negative attitudes toward COVID‐19 vaccination.

Variable	Number	% negative	aOR	95% CI	*p*
Sex					
Male	63	55.6	1.00	Reference	—
Female	179	40.2	1.67	0.96–2.91	0.071
Age group					
> 21–25 years	96	35.4	1.00	Reference	—
18–21 years	146	50.0	1.29	0.77–2.16	0.334
City of origin					
Elbasan	158	43.0	1.00	Reference	—
Other cities	84	46.4	1.14	0.66–1.96	0.641
Residence area					
Urban	167	33.5	1.00	Reference	—
Rural	75	68.0	2.64	1.51–4.61	0.001
Living arrangement					
With family	122	32.8	1.00	Reference	—
Dormitory	59	47.5	1.82	1.03–3.24	0.039
With friends	61	34.4	1.41	0.76–2.63	0.271
Degree					
Master's	88	34.1	1.00	Reference	—
Bachelor's	154	50.0	1.36	0.80–2.31	0.255

*Note:* Hosmer–Lemeshow *p* = 0.624; Nagelkerke *R* ^2^ = 0.19; AUC = 0.73 (95% CI 0.66–0.79). All VIF values were < 2.0.

Abbreviations: aOR, adjusted odds ratio; AUC, area under the curve; CI, confidence interval; VIF, variance inflation factor.

Multicollinearity diagnostics indicated no important collinearity among the predictors (all VIF values < 2.0). The model showed acceptable calibration according to the Hosmer–Lemeshow goodness‐of‐fit test (*p* = 0.624), modest explanatory performance (Nagelkerke *R* ^2^ = 0.19), and acceptable discrimination (AUC = 0.73, 95% CI 0.66–0.79) (Table 11).

**TABLE 11:** Sensitivity analyses using alternative thresholds for the dichotomized attitude outcome.

Variables	Main threshold aOR (95% CI)	*p*	Alternative threshold 1 aOR (95% CI)	*p*	Alternative threshold 2 aOR (95% CI)	*p*
Sex						
Male	1.00 (Reference)	—	1.00 (Reference)	—	1.00 (Reference)	—
Female	1.67 (0.96–2.91)	0.071	1.54 (0.90–2.64)	0.113	1.73 (0.88–3.39)	0.110
Age group						
> 21–25 years	1.00 (Reference)	—	1.00 (Reference)	—	1.00 (Reference)	—
18–21 years	1.29 (0.77–2.16)	0.334	1.21 (0.73–2.01)	0.458	1.36 (0.72–2.55)	0.344
City of origin						
Elbasan	1.00 (Reference)	—	1.00 (Reference)	—	1.00 (Reference)	—
Other cities	1.14 (0.66–1.96)	0.641	1.08 (0.63–1.85)	0.782	1.19 (0.61–2.31)	0.612
Residence area						
Urban	1.00 (Reference)	—	1.00 (Reference)	—	1.00 (Reference)	—
Rural	2.64 (1.51–4.61)	0.001	2.31 (1.34–3.98)	0.003	2.88 (1.42–5.84)	0.003
Living arrangement						
With family	1.00 (Reference)	—	1.00 (Reference)	—	1.00 (Reference)	—
Dormitory	1.82 (1.03–3.24)	0.039	1.69 (0.97–2.96)	0.064	1.94 (1.01–3.74)	0.047
With friends	1.41 (0.76–2.63)	0.271	1.33 (0.72–2.45)	0.360	1.46 (0.71–3.01)	0.302
Degree						
Master's	1.00 (Reference)	—	1.00 (Reference)	—	1.00 (Reference)	—
Bachelor's	1.36 (0.80–2.31)	0.255	1.29 (0.76–2.18)	0.347	1.41 (0.75–2.67)	0.286

*Note:* Alternative threshold 1 and Alternative threshold 2 represent sensitivity analyses based on different cut‐offs for the dichotomized attitude score. The same theory‐based covariates were retained in all models for comparability.

Sensitivity analysis excluding students vaccinated because of mandatory requirements:

A further sensitivity analysis was performed after excluding students who reported that they had been vaccinated primarily because vaccination was mandatory. After this exclusion, the direction of the main associations remained similar to that of the primary model. Rural residence remained associated with higher odds of negative attitudes toward COVID‐19 vaccination (aOR = 2.47, 95% CI 1.28–4.76, *p* = 0.007), and students living in dormitories also continued to show higher odds of negative attitudes compared with those living with family (aOR = 1.71, 95% CI 0.89–3.28, *p* = 0.107), although the estimate was less precise.

Associations for sex, age group, city of origin, and degree level remained weak and were not materially different from the primary analysis. These findings suggest that the main pattern of results was not driven solely by participants vaccinated under mandatory requirements (Table 12).

**TABLE 12:** Sensitivity analysis excluding students vaccinated because of mandatory requirements.

Variable	Primary model aOR (95% CI)	*p*	Excluding mandatory vaccinators aOR (95% CI)	*p*
Sex				
Male	1.00 (Reference)	—	1.00 (Reference)	—
Female	1.67 (0.96–2.91)	0.071	1.59 (0.84–3.00)	0.153
Age group				
> 21–25 years	1.00 (Reference)	—	1.00 (Reference)	—
18–21 years	1.29 (0.77–2.16)	0.334	1.24 (0.67–2.28)	0.490
City of origin				
Elbasan	1.00 (Reference)	—	1.00 (Reference)	—
Other cities	1.14 (0.66–1.96)	0.641	1.09 (0.58–2.05)	0.789
Residence area				
Urban	1.00 (Reference)	—	1.00 (Reference)	—
Rural	2.64 (1.51–4.61)	0.001	2.47 (1.28–4.76)	0.007
Living arrangement				
With family	1.00 (Reference)	—	1.00 (Reference)	—
Dormitory	1.82 (1.03–3.24)	0.039	1.71 (0.89–3.28)	0.107
With friends	1.41 (0.76–2.63)	0.271	1.36 (0.66–2.83)	0.401
Degree				
Master's	1.00 (Reference)	—	1.00 (Reference)	—
Bachelor's	1.36 (0.80–2.31)	0.255	1.29 (0.69–2.40)	0.425

*Note:* The sensitivity analysis excluded students who reported vaccination primarily because it was mandatory by the government or faculty. The same theory‐based covariates were retained as in the primary model.

## 4 Discussion

COVID‐19 has affected millions of people and put on difficulties in the healthcare systems throughout the world (Ramasaco et al. 2023) so the only hope to prevent this pandemic is through vaccines (Kashte et al. 2021). Di Giuseppe et al. (2021) argues that knowing if university students are willing to get vaccinated against COVID‐19 in the future and recognizing potential obstacles could assist public health officials in creating COVID‐19 containment plans and treatments that work (Di Giuseppe et al. 2021). The findings of a study conducted by Pouvrasseau and Jeannot (2023) are in the same line. They contend that knowledge of vaccine hesitancy among undergraduate nursing and midwifery students will enhance public health efforts by enabling targeted interventions and educational strategies to boost future healthcare professionals' confidence in vaccination (Pouvrasseau and Jeannot 2023).

Since December 11, 2020, when the Pfizer vaccine received the emergency use authorization, COVID‐19 vaccine production has been seen as a light at the end of the tunnel for all humanity. Beyond the progress that the COVID‐19 vaccine had and the public acceptance, the real challenge was its negative attitude concerning the vaccination. There are still skeptical people related to the undesirable side effects of the vaccine and its contribution to eradicating this disease. Furthermore, to this skepticism is added the reduction of protection that is created after vaccination and re‐infection of individuals with new variants of COVID‐19.

The Albanian government managed to provide the first vaccine doses on January 11, 2021, thus officially launching the anti‐COVID vaccination campaign ‘Albania Smiles’. The promotion and campaigns to invite people of all age categories to carry out the vaccination have been intense throughout the whole time. Although estimates of herd immunity and vaccination are changing rapidly, some of the estimates indicated at least 60% of a population needs to be vaccinated to achieve herd immunity (Mahmud et al. 2021). Based on the generical studies, it seems like a larger number of vaccines, approximately 570 million doses, are generated in the US (Suthar et al. 2022); moreover, more than 11 billion vaccine doses have been managed globally until 11 April 2022 (World Health Organization 2021b; CDC 2021). The World Health Organization's target is to vaccinate 70% of the world's population by mid‐2022 (World Health Organization 2022).

In Albania, the official data of the Ministry of Health show that only 52% of the population has been vaccinated (World Health Organization 2021a). Thus, the coverage rate of the population with the covid‐19 vaccine is low compared to the WHO target. In this study, we observed and presented the opinions and attitudes, and hesitancy concerning the vaccine against the COVID‐19 virus among students at the university. At the end of the summer, the Ministry of Health and the Ministry of Education issued an order regarding the vaccination campaign for students before they returned to the auditoriums in the faculties. Due to the very small number of vaccinated students in September, the two ministries made a series of promotional campaigns emphasizing the necessity of vaccination before the start of the 2021–2022 academic year. After this campaign at the beginning of October 2021, the most significant part of the students resulted to be vaccinated with one of the vaccines offered in our country. Thus, the rate of vaccination in student participants with almost one dose of the vaccine in this study resulted in 60.7%, while 27.7% of students had not been vaccinated and did not plan to do. However, certain students would probably be less willing to take the COVID‐19 vaccine if it were not required before the full academic semesters by the government or faculties.

George et al. (2022) highlighted in their study that COVID‐19 vaccine reluctance had jeopardized the achievement of immunization campaigns. Those researchers found that concerns about vaccine efficacy and vaccine‐related adverse events are possible impediments to immunization; nevertheless, it is unclear whether tailored messaging and vaccination programs might impact uptake (George et al. 2022).

The findings of this study have demonstrated that knowledge, perception, and beliefs about the COVID‐19 vaccine were over 50%. These percentage figures indicate a very strong willingness of students to take the vaccine, but at the same time, some might have disobedience to vaccination, or the feeling of fear regarding the production, the quick approval time of the vaccine, and the degree of vaccine coverage in front of new variants, etc. We must emphasize that this result is much lower than some other studies' findings. So, in one European study conducted by Neumann‐Böhme et al. (2020), the willingness to take the vaccine was reported at 73.9% in the general population (Neumann‐Böhme et al. 2020), and in another study conducted by Freeman et al. (2020) in the UK, the willingness was reported at 71.7% (Freeman et al. 2020), whereas 84.1% of participants in a different Di Giuseppe study stated that they would be willing to get the COVID‐19 vaccination in the future, despite some reservations about its efficacy and safety as well as the need for public health initiatives to reach the rate that can result in community protection (Di Giuseppe et al. 2021). Furthermore, the willingness to uptake the COVID‐19 vaccine among students in China and the USA resulted in being higher at 79.1% and 77%, respectively, compared to the rate of vaccination in our students (Bai et al. 2021; Lucia et al. 2020). In contrast to the results in Europe, our finding was upwards of the COVID‐19 vaccination acceptance rate among college students in Malta (44.2%) (Grech and Gauci 2020). Whereas in a study conducted by Uzochukwu et al. (2021) among staff and students in a Nigerian tertiary educational institution, approximately 34.70% of the university community were willing to apply the COVID‐19 vaccine at the time that the vaccine was supplied to them (Uzochukwu et al. 2021).

The interesting findings in this study were that 33.9% of the responders did not recognize vaccines as an effective tool for the pandemic, and 23.1% (56/242) think that COVID‐19 will be controlled only by preventive measures without vaccination. Additionally, 40.1% (97/242) think that vaccines have side effects such as causing infertility, 15.7% (38/242) think that vaccines can cause autism, Alzheimer's, or other illnesses, and 19% (46/242) think that vaccines permanently alter our DNA. Students who were not sure whether or not to be vaccinated were exposed to misinformation about the vaccine. Those have different attitudes that are related to the general lack of faith in vaccines. That is, when people encounter misinformation, they perceive less informational need for adequately preventing and treating COVID‐19. We must emphasize that exposure to misinformation reduced the information sufficiency threshold provided by health organizations and governments of each country. In regard to the safety and efficacy of vaccines, it will rival the misinformation provided on social media. The same findings of misinformation that resulted in this study were also reported in some previous research (Bai et al. 2021; Kim et al. 2020; Cordina et al. 2021; Wong et al. 2020). Because of this, it is advised in numerous works that educational initiatives and the dissemination of trustworthy information from reputable sources—primarily medical personnel—along with the accessibility of vaccinations, should be of a higher quality. In addition, it is imperative to distinguish between the descriptive prevalence of misinformation beliefs and the analytical outcomes related to attitudes. It is essential to establish a clear distinction between these aspects to avoid conflating neutral responses with positive endorsements, as this could lead to misinterpretations of the data. For instance, if a considerable proportion of respondents demonstrate neutral or indifferent attitudes toward misinformation, this should not be misinterpreted as a lack of concern or awareness. It is vital to emphasize these differences in order to facilitate a more nuanced understanding of the findings and their implications, which will ultimately aid in the design of more effective interventions.

In conclusion, whilst the present study provides valuable insights into the landscape of misinformation beliefs, it is imperative to exercise caution when considering its limitations and to formulate practical recommendations for informed decision‐making in the fields of communication and education. This emphasis on cautious interpretation underscores the importance of continued research in this area to develop a more comprehensive understanding of misinformation and its impact on society. Moreover, this would assist us in addressing vaccination reluctance and fostering effective herd immunity for the general public, including the young, the elderly, and those suffering from chronic illnesses (Sansone et al. 2024; Wang et al. 2021; Koja and Abazaj 2024).

The socio‐demographic characteristics among students related to the acceptance of the vaccines were associated with some factors. Females' immunity response is highly different from males in relation to many vaccines (Jensen et al. 2022). Our study shows that females were unlikely to believe the positive side effects of the vaccination compared to males. This lack of conviction by females is in the same line with findings in other studies (Galasso et al. 2020; Zintel et al. 2021). Females being afraid of injections seems like a non‐acceptance factor to taking the vaccination (Murphy et al. 2021).

Ages of 18 to 21 appear to be less positive to take the vaccine over those 21 years old. There was found an association between age and attitude concerning the vaccine. Szilagyi et al. have reported that educational background influenced their willingness to take the vaccine, especially those with a Bachelor'ss degree or higher who were skeptical believing the vaccination protection (Szilagyi et al. 2021). In our research, students at the Bachelor's level were less optimistic about taking the vaccination compared to them at the Master's level.

A clear view of students from urban areas compared to the rural ones shows us that those from urban areas positively support taking the vaccine Different mentality provides different attitudes. This difference was also found in previous studies (Zhai and Goss 2020; Yang et al. 2022). In cases where students were well informed of security and advocated for vaccination, receiving it was accepted as the optimal choice. This finding is similar to the conclusion of previous studies (Bai et al. 2021; Ma et al. 2018). If we make a resumé of all the findings in our study, it turns out that the key factors of the hesitation of getting the vaccine are directly linked to its safety and efficiency. The same situation results in several studies conducted on students in Canada and Europe (Verger et al. 2021; Bell et al. 2020; Wang et al. 2020). Furthermore, the presence of selection bias in the study is acknowledged due to certain exclusions in the sample; a limitation that could impede the generalizability of the findings to more extensive populations. For instance, if the sample predominantly comprised participants from a specific demographic or geographical area, this could result in the skewing of results and their applicability to other populations. This limitation must be considered when interpreting the results, as the sample may not fully represent the larger community. It is recommended that future studies adopt more inclusive and extensive sampling methods with a view to enhancing the generalizability of the findings.

In the adjusted theory‐based models, the most consistent associations were observed for residence area and living arrangement, whereas the associations for age group, degree level and city of origin were weaker after controlling for the other covariates. These findings should therefore be interpreted on the basis of the magnitude and precision of the estimates rather than statistical significance alone. The sensitivity analyses supported the robustness of the main findings. Similar directions of association were observed when alternative thresholds were used for the dichotomized attitude outcome, suggesting that the results were not driven solely by the selected cut‐off. Likewise, exclusion of students vaccinated primarily because of mandatory requirements did not materially alter the overall pattern of associations. However, a weighting‐based sensitivity analysis for non‐response could not be conducted because sufficiently detailed auxiliary information for the full invited population was unavailable; therefore, some degree of non‐response bias cannot be excluded.

The exploratory factor analysis further strengthened the psychometric interpretation of the adapted questionnaire. While Cronbach's alpha supported internal consistency, the factor analysis provided additional evidence that the composite domains reflected coherent underlying constructs in this sample. However, because the questionnaire was adapted from previous studies and applied in a specific Albanian university context, further validation in larger and more diverse student populations is warranted.

## 5 Strengths and Limitations

One of the study's strengths is that it recruited numerous students from six study programs in a short period of time. Furthermore, it was the country's first study based on students' thoughts and attitudes regarding immunization for COVID‐19.

On the other hand, there are various limitations to our study. First, this study was conducted during a tough moment when students were faced with a difficult choice: in order to return to classes, they must first be vaccinated.

The number of students involved in this study is relatively small, representing around one‐tenth of all students enrolled during the academic year when this study was accomplished.

Third, the questions were filled out by the students, so we do not know if the responses were correct, implying a lack of genuine recognition of the vaccine's COVID‐19 opinions, attitudes and hesitancy concerns among students.

Although response bias, missing data and construct validity were addressed through additional analyses, residual selection bias cannot be excluded because of the anonymous survey design and the exclusion of extensively incomplete questionnaires.

The cross‐sectional design of this study limits the cause‐and‐effect relationship. Another problem is that this study did not cover all students across the country, limiting its generalizability to other students across the country.

## 6 Conclusion

The present study employs a cross‐sectional design, a methodological framework that inherently restricts the capacity to derive causal inferences concerning the relationships between variables. The design under consideration provides a concise representation of the data at a specific moment in time. While it facilitates the examination of associations, it does not encompass the dynamic processes that may emerge over extended periods. The results of this detailed study show skepticism about the COVID‐19 vaccine among university students, even though the vaccination rate was 60.9%. How this unknown virus was treated, and scant information on vaccination, influenced distrust and fear in students.

All findings suggest that public education on the efficacy and safety of the COVID‐19 vaccine is important for the future widespread use of the vaccine. Vaccine administration programs should encourage students to choose to be vaccinated against COVID‐19. In regard to skeptical students about whether to get vaccinated, these programs need to provide knowledge with proper educational materials that dispel myths and misconceptions. As a consequence, barriers will diminish and also assist students in reaching an affirmative decision to participate in a future vaccination campaign.

In the context of the practical implications that have been derived from the findings of this study, it is imperative to consider these as suggestions rather than definitive conclusions. Specifically, the present study supports the theory that the implementation of enhanced communication strategies and educational initiatives could prove an effective means of addressing misinformation beliefs. The findings of this study suggest that individuals who are better informed might hold more resilient attitudes toward misinformation. Therefore, the development of targeted educational programs could be beneficial. However, it is imperative to emphasize that these suggestions are not conclusive, but rather represent avenues for further exploration. It is recommended that future research investigate the efficacy of specific communication strategies. Such strategies may include the use of fact‐checking websites, public awareness campaigns, or the role of trusted figures in dispelling misinformation.

## Author Contributions

Gjergji Koja and Erjona Abazaj were involved in the conception and design of the study; Gjergji Koja, Erjona Abazaj, Shpetim Qyra, and Ledina Nikolla were part of the data collection and data curation, analysed the data, and methodology, drafted the manuscript, and approved the manuscript. Gjergji Koja and Erjona Abazaj were responsible for supervision, validation, and visualization, writing the original drafts and review editing. Lila Shundi, Zahide Sulejmani, Ela Ali, Brunilda Hysaj, writing – review and editing the paper after the second review. Revising the work critically for important intellectual content and interpretation of data for the paper after the second revision. Final approval of the version to be published, and agreement to be accountable for all aspects of the work in ensuring that questions related to the accuracy are addressed.

## Funding

The authors have nothing to report.

## Ethics Statement

The Study Protocol was submitted to the Ethics Committee of the Alexander Xhuvani Faculty. Ethical clearance was obtained from the rector of the University, Alexander Xhuvani, Elbasan, Albania (issue date 14/09/2020). Due to technical difficulties caused by the pandemic situation, student participants in this survey were informed through verbal communication during the online classes. In this survey, no personal data were recorded, and all questionnaires were completed anonymously. Participation in the study was voluntary, and participants could withdraw at any moment. We warrant that all ethical guidelines for medical research are strictly respected.

## Consent

All authors have given their consent for the publication of this paper.

## Conflicts of Interest

The authors declare no conflicts of interest.

## Data Availability

The data that support the findings of this study are available from the corresponding author upon reasonable request.
